# *In Silico* Screening Identifies a Novel Potential PARP1 Inhibitor Targeting Synthetic Lethality in Cancer Treatment

**DOI:** 10.3390/ijms17020258

**Published:** 2016-02-19

**Authors:** Jian Li, Nan Zhou, Peiling Cai, Jinku Bao

**Affiliations:** 1 School of Medicine, Chengdu University, Chengdu 610106, China; caipeiling@cdu.edu.cn; 2 College of Life Sciences and Key Laboratory for Bio-Resources of Ministry of Education, Sichuan University, Chengdu 610064, China; zhnanx@live.com

**Keywords:** synthetic lethality, PARP inhibitor, DNA damage response (DDR)

## Abstract

Synthetic lethality describes situations in which defects in two different genes or pathways together result in cell death. This concept has been applied to drug development for cancer treatment, as represented by Poly (ADP-ribose) polymerase (PARPs) inhibitors. In the current study, we performed a computational screening to discover new PARP inhibitors. Among the 11,247 compounds analyzed, one natural product, ZINC67913374, stood out by its superior performance in the simulation analyses. Compared with the FDA approved PARP1 inhibitor, olaparib, our results demonstrated that the ZINC67913374 compound achieved a better grid score (−86.8) and amber score (−51.42). Molecular dynamics simulations suggested that the PARP1-ZINC67913374 complex was more stable than olaparib. The binding free energy for ZINC67913374 was −177.28 kJ/mol while that of olaparib was −159.16 kJ/mol. These results indicated ZINC67913374 bound to PARP1 with a higher affinity, which suggest ZINC67913374 has promising potential for cancer drug development.

## 1. Introduction

The concept of synthetic lethality has recently emerged in the field of cancer treatment. This concept was borrowed from classical genetics to describe situations in which defects in two different genes or pathways together result in cell death, while a defect in one of the two does not affect viability [1,2]. Because the deficiency of certain DNA damage response (DDR) gene(s) or pathway(s) are observed in virtually all types of cancer, drugs targeting the complementary pathway of the defective DDR function would be an ideal strategy for cancer treatment with desired high specificity [3].

Poly (ADP-ribose) polymerases (PARPs) inhibitors are a group of chemical compounds that are being developed for cancer treatment under the concept of synthetic lethality. PARPs are enzymes that transfer ADP-ribose moieties to a variety of protein substrates [4]. The physiological function of ADP-ribosylation is best characterized in the context of genome stability maintenance, in which ADP-ribose polymers facilitate the recruitment of the proteins to sites of DNA damage [4]. Inhibition of PARP-1 leads to the accumulation of single-strand breaks (SSBs) that are converted to double strand-strand breaks (DSBs) during DNA replication. The generated DSBs can be repaired either by homologous recombination (HR) or non-homologous end joining (NHEJ) [5,6]. The inhibition of PARP has been shown to be synthetically lethal with loss of BRCA1 and BRCA2, which play essential roles in HR-mediated DSB repair [7,8,9]. Moreover, defects in the DNA damage response proteins, such as NBS1, MRE11, ATR, ATM, FANCD2, FANCA, FANCC, Chk1, Chk2, and ERCC1, also confer selective sensitivity to PARP inhibition [10,11,12,13,14].

The therapeutic potential of PARP inhibitors may extend to a larger cohort of patients than initially indicated. It is of great significance to identify potent PARP1 inhibitors to target the complementary pathways in these synthetic lethal pairs. In the current study, we performed a computational screening to discover new PARP inhibitors with drug development potential. Among the 11,247 compounds analyzed, one natural product, ZINC67913374, was identified as a potential PARP1 inhibitor, because of its superior performance in the simulation analyses.

## 2. Results

### 2.1. Docking Performance

**Figure 1:**
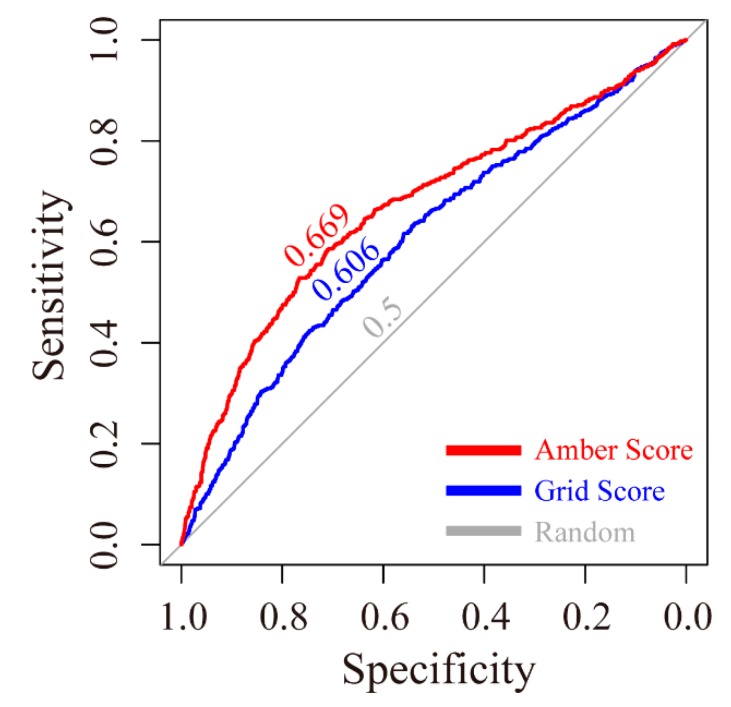
ROC evaluation of grid and amber scoring functions. Corresponding AUC values for each ROC curve are labeled above the line. Color code: Red—amber scoring function; Blue—grid scoring function; Gray—random.

The receiver operating characteristic (ROC) curves for the grid scoring and amber scoring functions were plotted. As shown in Figure 1, the area under curve (AUC) of grid score and amber score were 0.606 and 0.669, respectively. Both are higher than the random condition, which only gave a 0.500 AUC value.

### 2.2. Potential PARP1 Inhibitor

The reference drug, olaparib, achieved a grid score of −61.41 and amber score of −51.18 (Table 1), which was set as the cut-off values for selecting potential PARP1 inhibitors. Compared to olaparib, 631 out of 11,247 natural compounds received a higher grid score. These hits were rescored using the amber scoring function, and one compound, ZINC67913374, stood out with an amber score of −51.42 (Table 1). ZINC67913374 was therefore identified as a potential inhibitor against PARP1.

### 2.3. Binding Modes

**Figure 2:**
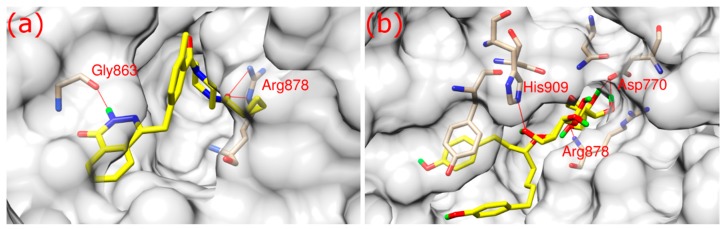
Binding modes of inhibitors towards PARP1 at the binding site. (**a**) Binding mode of olaparib; (**b**) binding mode of ZINC67913374. The surface of PARP1 is presented as gray with 70% transparency. Inhibitors are shown as yellow stick. Corresponding residues of PRAP1 forming hydrogen bonds with ligands are displayed as tan stick. Color code for elements: tan—C of PARP1; yellow—C of inhibitor; blue—N; red—O; green—H.

As shown in Figure 2, both olaparib and ZINC67913374 bound to PARP1 in its binding pocket. They interact with PARP1 through hydrophobic interaction and hydrogen bonds formation. Olaparib bound to PARP1 by forming three hydrogen bonds (Figure 2a): its O3 formed two hydrogen bonds with the NE and NH2 of Arg878 at a distance of 3.1 and 2.9 Å, respectively; N2 of olaparib formed another hydrogen bond with the O of Gly863 at a 2.8 Å distance.

ZINC67913374 formed four hydrogen bonds with PARP1 (Figure 2b). Similar to olaparib, the O9 of ZINC67913374 formed one hydrogen bond with the N of Arg878 at a distance of 3 Å. ZINC67913374 formed another hydrogen bond at 2.8 Å between its O4 and NE2 of His909. OD2 of ZINC67913374 formed two hydrogen bonds with Asp770, one with O10 (2.6 Å) and another with O11 (2.5 Å).

### 2.4. Stability of Receptor-Ligand Complex

**Figure 3:**
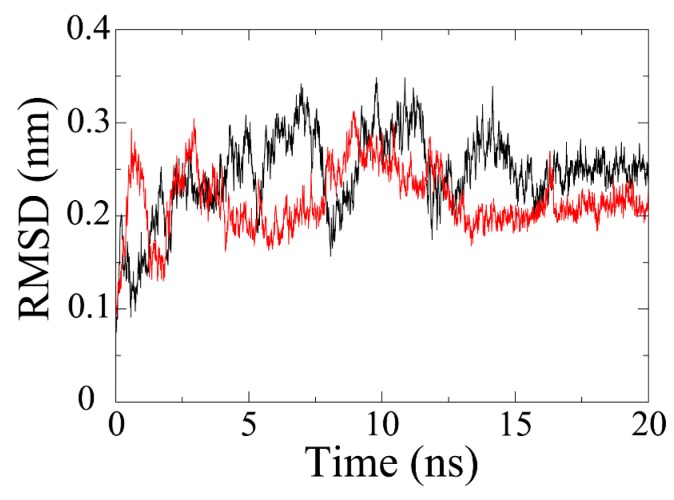
Backbone RMSD of PARP1^®^C inhibitor complexes. Black line denotes RMSD of the olaparib system while red line represents the PARP1-ZINC67913374 complex.

MD simulation is exploited to evaluate the stability of a protein-ligand system. As shown in Figure 3, the RMSD of olaparib-PARP1 complex has gradually increased to about 0.34 nm by 7 ns. Afterwards, it plunged to about 0.16 nm at 8 ns and then jumped to the highest point (0.35 nm) at around 10 ns. The RMSD value fluctuated in the following 6 ns and stabilized at 0.25 nm by 16 ns. In terms of PARP1-ZINC67913374 complex, the RMSD reached around 0.3 nm in just 1 ns. After that, it plummeted by 2 ns and then experienced a rise trend till 4 ns. Then, the RMSD fell and fluctuated at around 0.2 nm over the next 4 ns. It began fluctuating markedly at 8 ns. The equilibrium (0.2 nm) was reached by 14 ns, which lasted for 6 ns until the end of the simulation.

### 2.5. Binding Free Energy

The binding free energy of ZINC67913374 and olaparib towards PARP1 were listed in Table 2. Compared with olaparib (−159.16 kJ/mol), ZINC67913374 (−177.28 kJ/mol) had a smaller binding energy value. For both inhibitors, their gas-phase contribution, namely the combination of van der Waals energy and electrostatic energy, was favorable for inhibitor binding. Their solvation contribution was positive and unfavorable, which may result from strong unfavorable polar energy and weak favorable nonpolar energy. After scrutinizing the four contributing factors, we found both inhibitors had similar van der Waals energy (−293.07 kJ/mol for ZINC67913374 and −210.36 kJ/mol for olaparib) and nonpolar contribution (−29.22 kJ/mol for ZINC67913374 and −21.09 kJ/mol for olaparib). However, remarkable differences were observed in electrostatic energy (−327.42 kJ/mol for ZINC67913374 and −89.19 kJ/mol for olaparib) and polar solvation energy (472.47 kJ/mol for ZINC67913374 and 161.49 kJ/mol for olaparib).

**Figure 4:**
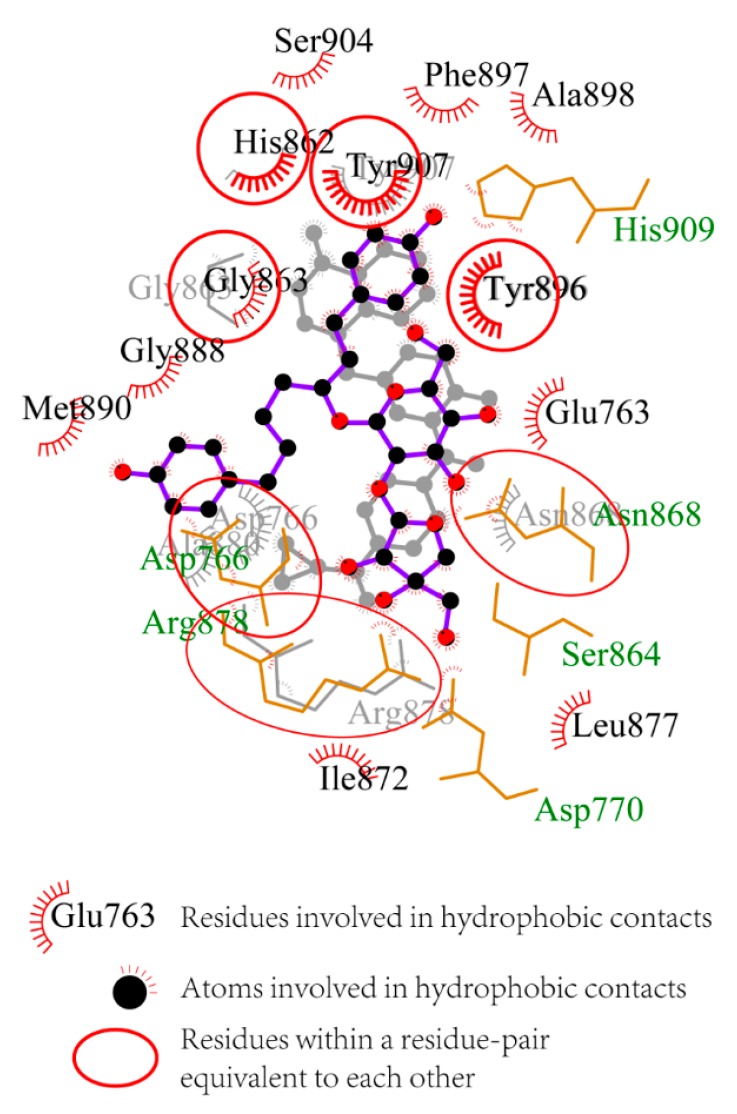
Superimposed 2D interaction diagrams of olaparib (background) and ZINC67913374 (foreground) with PARP1. Ball and stick denotes ligands. Corresponding PARP1 residues are shown as wires.

The 2D interaction diagrams generated by LigPlot+ for olaparib- and ZINC67913374-PARP1 complexes were presented in Figure 4 [15]. The two complexes are fairly similar, so that they can be superimposed on each other. The superposition of the two related diagrams highlighted conserved interactions within olaparib- and ZINC67913374-PARP1 complexes. As shown in Figure 4, His862, Tyr907, Tyr896, Aan868, Arg878, Asp766, and Gly863 were conserved residues. Gly863 and Tyr907 had been reported as key amino acid residues for inhibitors–PARP1 interactions [16]. Gly863 participated in hydrogen bonding interaction network, while Tyr907 is involved in pi-pi stacking. Tyr907 was a key residue, which was not identified in olaparib–PARP1 interaction, but in the interaction between ZINC67913374 and PARP1. The other residues, including Ser904, Phe897, Ala898, Glu763, Leu877, Ile872, Met890, and Gly888, interacted with PARP1 via hydrophobic contacts. All the data above supported a stronger interaction between ZINC67913374 and PARP1, compared with the olaparib–PARP1 interaction.

**Figure 5:**
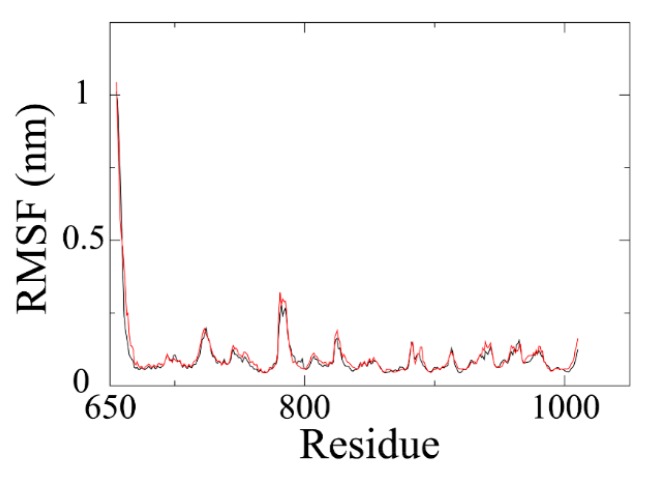
RMSF plots of backbone atoms for PARP1^®^Cinhibitor systems. Black line is for olaparib and red line is the PARP1-ZINC67913374 system.

**Figure 6:**
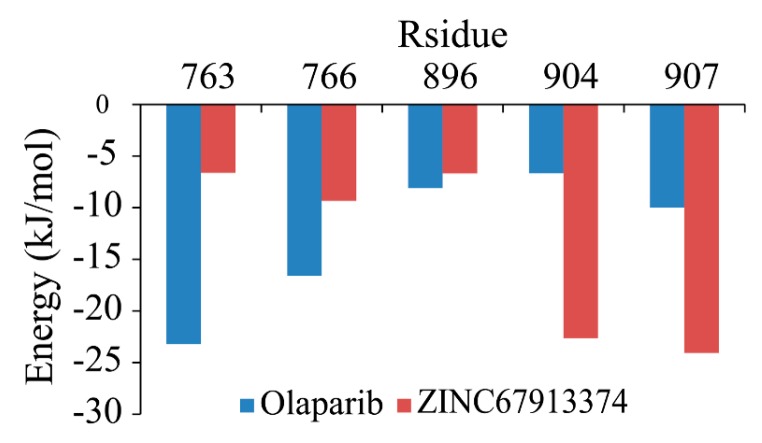
Binding free energy decomposition on a per-residue basis for olaparib- and ZINC67913374-PARP1 complexes.

Backbone RMSF (root mean square fluctuation) of PARP1 within ZINC67913374-PARP1 complex was comparable to that of the olaparib-PARP1 system (Figure 5). We scrutinized the contribution of each residue in the receptor–ligand interaction by binding energy decomposition (Figure 6). In both PARP1-inhibitor complexes, Glu763, Asp766, Tyr896, Ser904, and Tyr907 were critical residues for the binding interaction. This observation was consistent with the result that ZINC67913374 and olaparib share similar features on the RMSF profile when binding to PARP1.

### 2.6. ADMET Analysis

We predicted ZINC67913374’s ADMET using admetSAR, a free tool for evaluating chemical ADMET properties [17]. The result (Table S1) shows even with >500 molecular weight, ZINC67913374 can permeate the blood–brain barrier. The prediction also shows it has no AMES toxicity and no carcinogenicity.

## 3. Discussion

Currently, the majority of cancer therapies target proliferating cells rather than cancer cells *per se*. Cancer cells with a low proliferative index may evade treatment, while highly proliferative normal cells are also attacked by the same therapies. Taking advantage of the dysregulated DNA damage response in cancer using the synthetic lethality approach may be one of the most promising prospects for the future of cancer treatment. Similar to ovarian and breast cancers, some sporadic prostate, pancreatic and other tumors also possess DNA damage response defects due to mutation or epigenetic inactivation of HR components, suggesting that PARP inhibitors might be more broadly applicable.

By computational approaches integrating virtual screening, molecular dynamics simulation, binding free energy calculation and decomposition, we discovered a new potential inhibitor for PARP1 from natural products. Compared to the currently FDA-approved olaparib, this chemical exhibited a higher binding affinity to PARP1. It is projected to result in dysregulation of DNA damage repair, indicating its therapeutic potential for the treatment of cancers. Our results demonstrated that ZINC67913374 has promising potential as a PARP1 inhibitor for cancer drug development. Song and colleagues described the identification of four PARP1 inhibitors from a large number of natural products by *in silico* screening and *in vitro* enzymatic assay [18]. The screening performed in the current study focused on the AnalytiCon Discovery NP database. This database has a high percentage of unique compounds compared with other databases, which should theoretically increase the chance for discovering new potential PARP inhibitors. Certainly, the physiological activity of this compound needs to be validated with *in vitro* and *in vivo* studies in the next stage of the study. On the other hand, it is imperative to report this intriguing result of the researchers work in the field. A greater understanding of the basis of PARP inhibitor response is required for translational and clinical development of these agents, and in order to establish which patients may derive the most therapeutic benefit from this class of inhibitors.

## 4. Materials and Methods

### 4.1. Structure Preparation

The 3D structure of PARP1 was downloaded from the RCSB Protein Data Bank (PDB) using accession number 4UND [19], in which PARP1 is in complex with a known inhibitor named BMN673.This structure is used for identifying the binding site of PARP1. The molecular graphics of PARP1 were prepared and analyzed with the UCSF Chimera package [20]. In this process, (i) solvent and non-complexed ions were removed from PARP1; and (ii) hydrogens and charges (of amber ff99sb force field) were added to the protein.

The molecule library of natural compounds from AnalytiCon Discovery NP, containing 11,247 ZINC entries, was downloaded on 25 July 2015 from the ZINC database [21]. All these natural products have been filtered according to the criteria from ZINC and are provided in ready-to-dock, 3D formats.

On 19 December 2014, the U.S. Food and Drug Administration approved olaparib capsules (Lynparza, AstraZeneca Pharmaceuticals LP) as a monotherapy for advanced ovarian cancer after treatment with three or more prior lines of chemotherapy. The FDA approved olaparib was used as a reference drug for selecting hits and candidate PARP1 inhibitors.

### 4.2. Dock and Virtual Screening

For the sake of discovering new PARP1 inhibitors with drug development potential, a virtual screening was carried out by docking natural compounds to the binding site of PARP1 using UCSF DOCK 6 [22]. The known inhibitor, olaparib, was used as a reference drug to select hits. The DOCK suite of programs first docked all the natural compounds to PARP1 by assigning them grid scores that represent to what extent a given ligand would bind to a specific target. The scores were compared with that of olaparib. The compounds with higher scores were selected as hits for the second round of screening. Subsequently, the hit compounds were rescored by DOCK amber rescoring function, which allows small structural rearrangements to reproduce the so-called induced fit while performing docking. The compounds achieved higher amber scores than that of olaparib were chosen as candidate PARP1 inhibitors.

The specific running parameters used in our study for DOCK were: (1) change probe radius was 1.0; (2) maximum sphere radius was 3.0 Å; (3) minimum sphere radius was 1.0 Å; (4) the radius used to select binding site from the position where the known olaparib bound to was 8.0 Å; and (5) extra margin enclosed in all 6 directions was 3.0.

### 4.3. Dock Protocol Evaluation

The ROC curve was employed to illustrate the performance of DOCK. For binary classification, the possible outcomes fall into four categories: true positive (TP, a prediction is positive and the actual value is also positive), false positive (FP, a prediction is positive but the actual value is negative), true negative (TN, both the prediction outcome and actual value are negative), and false negative (FN, the prediction outcome is negative while the actual value is positive). The true positive rate (TPR) known as sensitivity can be expressed as [23]

TPR = sensitivity = TP/(TP + FN)

The false positive rate (FPR) is also known as the fall-out and can be calculated as

FPR = 1− specificity = 1 − TN/(TN + FP)

At the beginning, actives (positive PARP1 inhibitors) and decoys (negative PARP1 inhibitors) were downloaded (on 25 July 2015) from the DUD-E database, which provides active compounds and challenging decoys for molecular docking programs [24]. 742 actives and 3710 decoys were docked to the binding site of PARP1. These compounds were sequentially analyzed by grid scoring and amber scoring. pROC library within R was used to plot ROC curves (by plotting the sensitivity against the specificity at various threshold settings) and calculate values of area under the ROC curve (AUC), by which the performance of DOCK can be evaluated quantitatively [25].

### 4.4. MD Simulation

MD simulations were performed using GROMACS 4.5 [26] package and amber ff99sb force field [27] with TIP3P water model [28]. Particle Mesh Ewald (PME) [29] was exploited to consider the long-range electrostatic interactions and the Linear Constraint Solver (LINCS) [30] algorithm was used to constrain bonds. The receptor-ligand complexes were solvated in a dodecahedron box of water, with a distance of 1.0 between the solute and the box. All systems were neutralized by adding Na^+^ and Cl^−^ at 0.15 mol/L. Before MD simulations, the complexes were relaxed to <1000 kJ/mol/nm by up to 50,000 cycles of steep descent minimization. After energy minimization, temperature of the system was controlled in the NVT (constant number of particles, volume, and temperature) ensemble to 300 K over 100 ps. The 100 ps NPT (constant number of particles, pressure, and temperature) equilibration was then performed with a reference pressure of 1 bar. After that, 20 ns MD simulations were performed with a time step of 2 fs and the coordinates of the complexes were saved every 8 ps.

### 4.5. Free Energy Calculation and Decomposition

Molecular mechanics Poisson-Boltzmann surface area (MM-PBSA) was applied as a scoring function in computational drug design to estimate the interaction free energies in biomolecular interactions [31]. The binding free energy of a protein-ligand system in solvent can be given by [32]:

ΔG_bind_ = G_complex_ − (G_protein_ + G_ligand_)

where Gcomplex is the total free energy of the protein-ligand complex and Gprotein and Gligand represent free energies of the isolate protein and ligand, respectively.

In this study, the GROMACS tool g_mmpbsa was used to calculate the binding free energy of the protein with ligand [33]. The MM-PBSA approach was used to calculate the binding free energy as follows [33,34]:

ΔG_bind_ = ΔE_gas_ + ΔG_solv_ = ΔE_vdw_ + ΔE_ele_ + ΔG_polar_ + ΔG_nonpolar_

ΔE_gas_ is the average molecular mechanics potential energy in a vacuum (*i.e.*, gas-phase energy), which includes van der Waals (ΔE_vdw_) and electrostatic (ΔE_ele_) interactions; ΔG_solv_ denotes contribution to the solvation free energy that consists of polar solvation (ΔG_polar_) and nonpolar solvation (ΔG_nonpolar_) energies.

Using the g_mmpbsa, the binding free energy of protein-ligand complex was calculated from 11 snapshots extracted every 0.2 ns from the 18 to 20 ns MD trajectory. Furthermore, the binding energy was decomposed on a per residue basis to analyze the individual energy contributions of each residue to the protein-ligand interaction.

## 5. Conclusions

ZINC67913374 identified in this study is a potent PARP1 inhibitor with drug development potential, as reveal by the extensive bioinformatics simulation analyses. The FDA approval of olaparib and other on-going PARP1 inhibition related clinical trials validates synthetic lethality as an effective therapeutic strategy in cancer drug development. The success of PARP1 inhibitors encourages the characterization and targeting of other synthetic lethality pairs in DNA damage response and repair pathways.

## Figures and Tables

**Table 1:** Candidate PARP1 inhibitors from virtual screening.

Compound	Structure	Grid Score	Amber Score
ZINC67913374	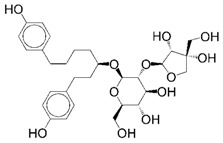	−86.8	−51.42
Olaparib	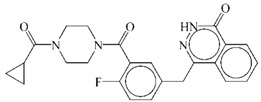	−61.41	−51.18

**Table 2:** Binding free energy (kJ/mol) of the potential PARP1 inhibitor and olaparib.

Inhibitor Components ^a^	ZINC67913374	Olaparib
ΔE_vdw_	−293.07 ± 10.25	−210.36 ± 11.77
ΔE_ele_	−327.42 ± 27.34	−89.19 ± 15.22
ΔG_ploar_	472.47 ± 28.21	161.49 ± 13.88
ΔG_nonpolar_	−29.22 ± 0.92	−21.09 ± 0.63
ΔG_bind_	−177.24 ± 24.78	−159.16 ± 15.13

^a^ ΔE_vdw_, van der Waals energy; ΔE_ele_, electrostatic contribution; gas-phase energy consists of ΔE_vdw_ and ΔE_ele_; ΔG_polar_, polar solvation energy; ΔG_nonpolar_, nonpolar solvation energy; the solvation free energy is a sum of ΔG_polar_ and ΔG_nonpolar_; ΔG_bind_, binding energy; ΔG_bind_ = ΔE_vdw_ + ΔE_ele_ + ΔG_polar_ + ΔG_nonpolar_.

## Supplementary Materials

Supplementary materials can be found at http://www.mdpi.com/1422-0067/17/2/258/s1.

## Abbreviations

DDR: DNA damage response
PARP: Poly (ADP-ribose) polymerase
SSB: single-strand break
DSB: double strand-strand break
HR: homologous recombination
NHEJ: non-homologous end joining
LINCS: Linear Constraint Solver
MM-PBSA: Molecular mechanics Poisson−Boltzmann surface area

## Appendix

**Table S1:** ADMET properties of ZINC67913374.

Property	Value	Prabability
Blood-Brain Barrier	BBB+	0.7028
Human Intestinal Absorption	HIA−	0.7104
Caco-2 Permeability	Caco2−	0.824
P-glycoprotein Substrate	Substrate	0.6847
P-glycoprotein Inhibitor (I)	Non-inhibitor	0.7057
P-glycoprotein Inhibitor (II)	Non-inhibitor	0.7339
Renal Organic Cation Transporter	Non-inhibitor	0.7435
CYP450 2C9 Substrate	Non-substrate	0.8328
CYP450 2D6 Substrate	Non-substrate	0.8277
CYP450 3A4 Substrate	Non-substrate	0.5479
CYP450 1A2 Inhibitor	Non-inhibitor	0.9058
CYP450 2C9 Inhibitor	Non-inhibitor	0.8699
CYP450 2D6 Inhibitor	Non-inhibitor	0.8892
CYP450 2C19 Inhibitor	Non-inhibitor	0.8244
CYP450 3A4 Inhibitor	Non-inhibitor	0.9023
CYP Inhibitory Promiscuity	Low CYP Inhibitory Promiscuity	0.8839
Human ERG Inhibition (I)	Weak inhibitor	0.8461
Human ERG Inhibition (II)	Inhibitor	0.5092
AMES Toxicity	Non AMES toxic	0.9003
Carcinogens	Non-carcinogens	0.9566
Fish Toxicity	High FHMT	0.9056
Tetrahymena Pyriformis Toxicity	High TPT	0.9662
Honey Bee Toxicity	High HBT	0.6725
Biodegradation	Not ready biodegradable	0.9337
Acute Oral Toxicity	III	0.5751
Carcinogenicity (Three-class)	Non-required	0.5401
Model	Value	Unit
Aqueous solubility	−1.3682	LogS
Caco-2 Permeability	−0.5376	LogPapp, cm/s
Rat Acute Toxicity	2.5494	LD50, mol/kg
Fish Toxicity	1.8239	pLC50, mg/L
Tetrahymena Pyriformis Toxicity	0.4575	pIGC50, ug/L
